# Checkerboard Helmholtz Resonator Metasurface for Dual-Mode Decoupled Dual-Band Coherent Perfect Absorption with Independently Tunable Frequencies

**DOI:** 10.3390/mi17040406

**Published:** 2026-03-26

**Authors:** Zimou Liu, Wenbo Liu, Zikai Du, Rui Yang

**Affiliations:** 1 National Key Laboratory of Radar Detection and Sensing, School of Electronic Engineering, Xidian University, Xi’an 710071, China; 23021211948@stu.xidian.edu.cn (Z.L.); 21021221357@stu.xidian.edu.cn (W.L.); ilvyzz@163.com (Z.D.); 2 China Academy of Space Technology (Xi’an), Xi’an 710000, China

**Keywords:** checkerboard Helmholtz resonator metasurface, coherent perfect absorption, dual mode, dual band, tunable

## Abstract

We present a checkerboard metasurface integrating interleaved Helmholtz resonator arrays with distinct geometrical parameters, enabling decoupled dual-band coherent perfect absorption (CPA) in both in-phase and anti-phase excitation conditions. Full-wave simulations confirm that the proposed structure achieves absorption rates exceeding 99% at 2.904, 3.024, 3.788 and 3.856 THz, corresponding to two pairs of resonant modes enabled by the asymmetric transmission characteristics. Notably, by actively manipulating the relative phase difference between the two excitation modes, the absorption frequencies associated with each CPA channel can be independently and continuously tuned. Benefiting from the planar checkerboard configuration, which combines compact geometry, suppressed mutual coupling, and balanced energy distribution, the metasurface achieves stable and independent dual-band absorption characteristics. The proposed design provides a promising pathway for the development of terahertz coherent absorbers with enhanced frequency stability and spectral flexibility of dual-mode operations, offering strong potential for practical photonic and electromagnetic applications.

## 1. Introduction

Helmholtz resonators (HRs) have been extensively utilized in acoustic systems for sound absorption, noise control, and energy dissipation due to their simple geometry and strong local resonance [1,2]. In recent years, the concept of Helmholtz resonance has been successfully extended from acoustics to electromagnetic (EM) domains, where HR-inspired structures have been employed to realize microwave and terahertz absorbers with compact size and efficient dissipation mechanisms [3]. These studies have demonstrated the feasibility of Helmholtz resonator metasurfaces as promising candidates for low-profile electromagnetic absorbers for predefined operating frequencies. However, coherent perfect absorption (CPA) based on HRs remains relatively unexplored. Conventional HR-based absorbers intrinsically exhibit highly localized, point-frequency resonant responses due to the lumped acoustic–electromagnetic nature of Helmholtz cavities and are therefore predominantly designed for unidirectional or incoherent illumination scenarios [4].

In such configurations, absorption originates from intrinsic material losses and impedance matching, leading to unidirectional perfect absorption (UPA) at isolated resonance frequencies. In contrast, CPA requires the coherent interference of counter-propagating waves, enabling complete energy trapping through phase-controlled excitation [5,6]. Although phase modulation in CPA systems provides an additional degree of freedom for tuning absorption amplitude, most reported HR-based CPA structures still operate at fixed resonance frequencies, where varying the relative phase between incident waves mainly modulates the absorption efficiency rather than the spectral position of the absorption peak [7]. Consequently, the inherent narrowband and point-frequency nature of HR resonances remains largely unaltered, significantly limiting their spectral flexibility and functional adaptability.

Furthermore, existing approaches to multi-frequency HR absorbers typically rely on multilayer stacking or cascaded resonator configurations to introduce multiple resonant channels [8,9,10,11]. While effective in extending the number of absorption bands, such designs inevitably increase structural thickness and induce strong inter-element coupling, often resulting in spectral instability and degraded absorption performance due to parasitic near-field interactions. These drawbacks pose serious challenges for the realization of compact, stable, and phase-sensitive coherent absorption platforms. To overcome these limitations, we propose a planar checkerboard HR metasurface, composed of interleaved HR units with distinct geometrical parameters. Beyond merely supporting multiple resonant frequencies, the proposed architecture enables phase difference-assisted modulation of the absorption frequency itself, allowing the absorption peaks associated with both in-phase and anti-phase CPA modes to be continuously shifted while maintaining absorption levels exceeding 80%. This mechanism effectively transforms the conventional point-frequency response of HR absorbers into a dynamically broadened absorption band, without resorting to multilayer stacking or increased thickness. Meanwhile, the checkerboard configuration suppresses mutual coupling and ensures balanced energy distribution.

## 2. Modeling and Numerical Results

**Figure 1:**
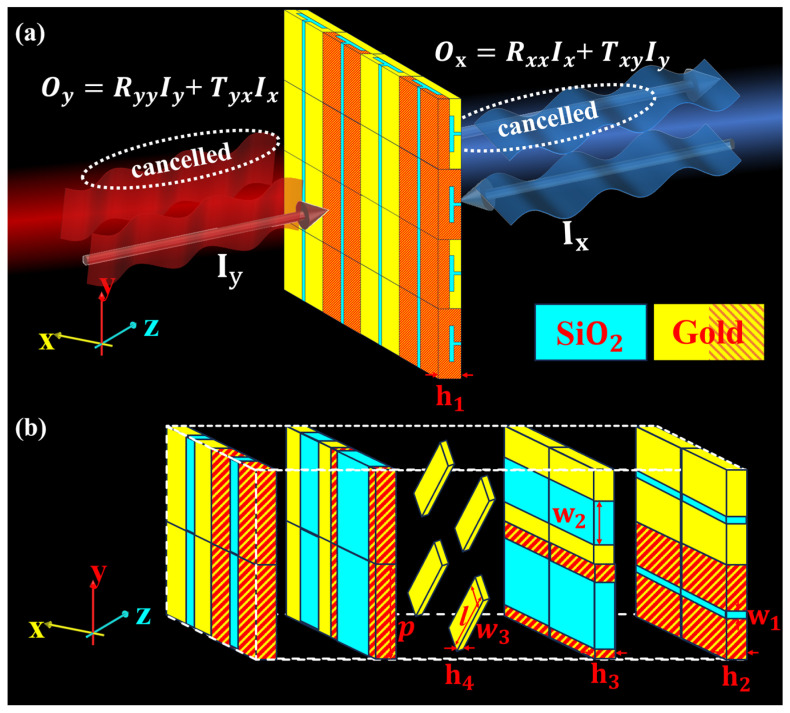
(**a**) Schematic diagram of the CPA based on the proposed checkerboard HR metasurface under the incidences with two different polarizations. (**b**) Structural parameters of the unit. The blue part represents silica dielectric with a relative permittivity of 3.9, and the metal gold is represented by the golden and red stripes for distinguishing resonant cavities of different dimensions.

Figure 1a illustrates the interaction between the checkerboard HR metasurface and electromagnetic fields with two different polarizations. Part of the incidences of $I_{x}$ ($I_{y}$) transmit into the HR, where it is converted into cross-polarized waves of $T_{yx}I_{x}\;(T_{xy}I_{y})$ by the 45°-tilted middle-layer metal block. The other part of the incidences will be reflected by the HR metasurface as $R_{xx}I_{x}$ ($R_{yy}I_{y}$). To achieve CPA, it is necessary for the output fields to be

(1) $$\left\{\begin{array}{c} O_{x}=R_{xx}I_{x}+T_{xy}I_{y}=0\\ O_{x}=R_{yy}I_{y}+T_{yx}I_{x}=0 \end{array}\right.$$

The metasurface employs a unit cell structure composed of four sub-units, formed by periodically arranging cavities of two different widths in both directions, as illustrated in Figure 1b, thereby achieving critical coupling absorption (CPA) at two distinct frequency points under two modes. The geometric parameters are as follows: the height of the entire unit $h_{1}=6\;\mu m$, unit period p=10 μm, $h_{2}=1.5\;\mu m$, resonator height $h_{3}=1.5\;\mu m$, slit width $w_{1}=0.4\;\mu m$, resonator width $w_{2}=5\;\mu m$, $w_{4}=7.5\;\mu m$, and the width $w_{3}=2.5\;\mu m$, thickness $h_{4}=0.7\;\mu m$, and length l=11.5 μm of the middle-layer metal block. For units on one diagonal, the upper and lower cavities have the same size, with both $w_{2}$ = 5 μm or $w_{4}$ = 7.5 μm. For cavities on the other diagonal, the widths of the upper and lower layers are different. If the width of the upper cavity is $w_{2}$ = 5 μm, shown as golden, the width of the orthogonal lower cavity is $w_{4}$ = 7.5 μm, and vice versa, shown as golden and red. Notably, gold for the 45°-tilted middle-layer metal block is modeled as a lossy metal with an electrical conductivity of ***σ*** = 4.561 × 10 ^7^ S/m and relative permeability *μ* = 1 in the 2.5~4.5 THz range, and its nearly frequency-independent conductivity with gently varying real and imaginary parts of the dielectric constant renders the dispersion effect negligible herein. The dielectric layer SiO_2_ (Corning 7980, Corning Incorporated, Corning, NY, USA) has a relative permittivity of $\varepsilon^{\prime }=3.9$ and a loss tangent of tanδ=0.002, which was measured over the 0.3–4 THz range. The proposed checkerboard Helmholtz resonator metasurface can be readily fabricated using standard micro/nanofabrication techniques for terahertz applications through a layer-by-layer process: first, the bottom metallic layer is deposited on an SiO_2_ substrate via electron-beam evaporation or sputtering and patterned using photolithography and lift-off. Second, the dielectric spacer layer is formed through spin-coating or CVD with precise thickness control. Third, the critical 45°-tilted middle metal layer can be implemented using grayscale lithography, multi-step alignment techniques, or approximated by a stepped structure, which is a well-established approach in terahertz metasurface fabrication. Finally, the top layer is completed through aligned lithography to establish the checkerboard configuration. Importantly, our design avoids complex multilayer resonator stacking and intricate 3D curved geometries that typically complicate fabrication, while maintaining feature sizes well within the resolution limits of conventional photolithography and electron-beam lithography systems, thus offering excellent manufacturability with current technologies.

**Figure 2:**
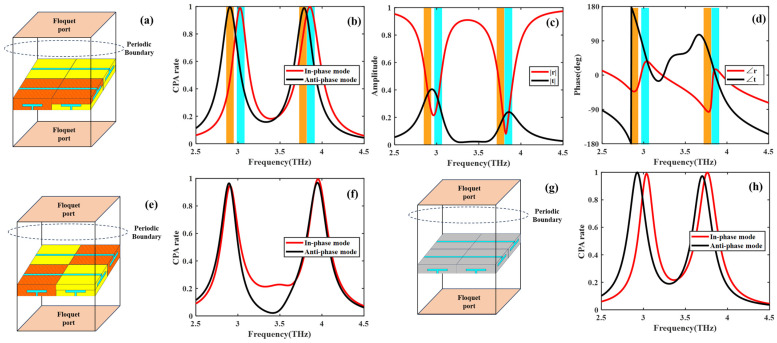
(**a**) Floquet mode analysis of the proposed checkerboard HR metasurface. (**b**) Absorption characteristics under in-phase and anti-phase excitation modes. (**c**) Amplitudes of transmission and reflection coefficients. (**d**) Phases of transmission and reflection coefficients. (**e**) Floquet mode analysis of the checkerboard-arranged HRs with alternating wide and narrow cavities. (**f**) Absorption characteristics under in-phase and anti-phase excitation modes. (**g**) Floquet mode analysis of the proposed checkerboard HR metasurface with non-destructive materials (PEC shown as gray; SiO_2_ without electrical conductivity shown as blue). (**h**) Absorption characteristics under in-phase and anti-phase excitation modes.

To verify the proposed design, full-wave simulations (CST Studio Suite) are performed. Figure 2a shows the Floquet mode analysis under periodic boundary conditions: the units are arranged periodically along the *x* and *y* directions, and the +*z* and −*z* directions are set as vacuum. The CPA rate is expressed as [12,13]:

(2) $$A_{c}=1-{\left(|r|-|t|\right)}^{2}-2|r||t|(1\pm \cos(∠r-∠t))$$

where *r* and *t* correspond to two sets of parameters—($R_{xx}$, $T_{xy}$) and ($R_{yy}$, $T_{yx}$)—which represent the coherent absorption parameters for the +*z* and −*z* incident directions. |r| and ∠r are the magnitude and phase of the reflection coefficient for a wave incident from one side, while |t| and ∠t are the magnitude and phase of the transmission coefficient for a wave incident from the other side. The asymmetrical transmission in such a design contributes to an additional mode. The “±” notation corresponds to the excitation mode: “+” for the in-phase mode and “−” for the anti-phase mode.

From the CPA rate results in Figure 2b, a CPA rate of 99.00% is achieved at 3.024 THz, 99.82% at 3.856 THz, 99.01% at 2.904 THz, and 99.77% at 3.788 THz. Among these, 3.024 THz and 3.856 THz correspond to in-phase mode coherent absorption, while 2.904 THz and 3.788 THz correspond to anti-phase mode coherent absorption. From Figure 2c,d, the metasurface exhibits near-equal reflection and transmission magnitudes as well as near-equal phase differences, satisfying the condition for the in-phase mode. Specifically, at 3.024 THz, the reflection and transmission coefficients are characterized by |r| = 0.3687 and |t| = 0.3218, with the phase angles of ∠r = 36.39° and ∠t = 48.42°, respectively. Similarly, at 3.856 THz, we have |r| = 0.2250, |t| = 0.2391, ∠r = 14.90°, and ∠t = 4.97°. In both cases, the phase difference between the reflected and transmitted waves approaches 0°. This phase-matching condition satisfies the in-phase CPA at these frequencies. Notably, the 45°-tilted middle-layer metal block endows the metasurface with polarization conversion capability, which assists in fulfilling the conditions of $\left|R_{xx}I_{x}\right|=\left|T_{xy}I_{y}\right|,\left|∠R_{xx}I_{x}-∠T_{xy}I_{y}\right|=0/180{}^{\circ}$ or $\left|R_{yy}I_{y}\right|=\left|T_{yx}I_{x}\right|,\left|∠R_{yy}I_{y}-∠T_{yx}I_{x}\right|=0/180{}^{\circ}$. This capability serves as the key to achieving CPA under both excitation modes. Under anti-phase excitation, the metasurface fulfills the CPA condition through an opposite phase relationship. At 2.904 THz, the extracted parameters are |r| = 0.2138, |t| = 0.1925, ∠r = −100.24°, and ∠t = 56.31°. Meanwhile, at 3.7880 THz, we have $\left|r\right|=0.3492,$ |t| = 0.3836, ∠r = −41.32°, and ∠t = 143.93°. In these cases, the reflected and transmitted waves exhibit a phase difference close to 180°. This phase opposition leads to destructive interference in the output channels under the anti-phase mode excitation, effectively suppressing both reflection and transmission and thus giving rise to CPA. By leveraging the polarization conversion capability of the 45°-tilted metal block and regulating the relative phase difference of incident waves to 0° for in-phase mode or 180° for anti-phase mode, the proposed metasurface achieves four distinct perfect absorption frequency points (2.904 THz, 3.024 THz, 3.7880 THz, and 3.856 THz) corresponding to two pairs of CPA modes. This represents a doubling of perfect absorption frequency points compared to traditional single-mode coherent absorbers, which typically only support one set of CPA conditions of anti-phase excitation and a limited number of perfect absorption frequencies. Subsequently, a comparative analysis is conducted between the absorption performance of the proposed checkerboard HR metasurface and that of a conventional metasurface solely comprising checkerboard-arranged HRs with alternating wide and narrow cavities. As illustrated in Figure 2e,f, the latter configuration presents the unit cell schematic and corresponding absorption spectra, exhibiting CPA exclusively at 2.896 THz and 3.952 THz. Consequently, discerning between the two resonant modes in this conventional design lacks physical significance. In stark contrast, the distinct spectral separation of the dual absorption modes achieved by the proposed metasurface will prove critically important for the subsequent investigation presented in this work. Meanwhile, we conducted simulations on the influence of the loss tangent. As shown in Figure 2g,h, in the absence of the loss tangent, the maximum peak shift is 0.032 THz, and the maximum reduction in the CPA rate peak is 0.008.

**Figure 3:**
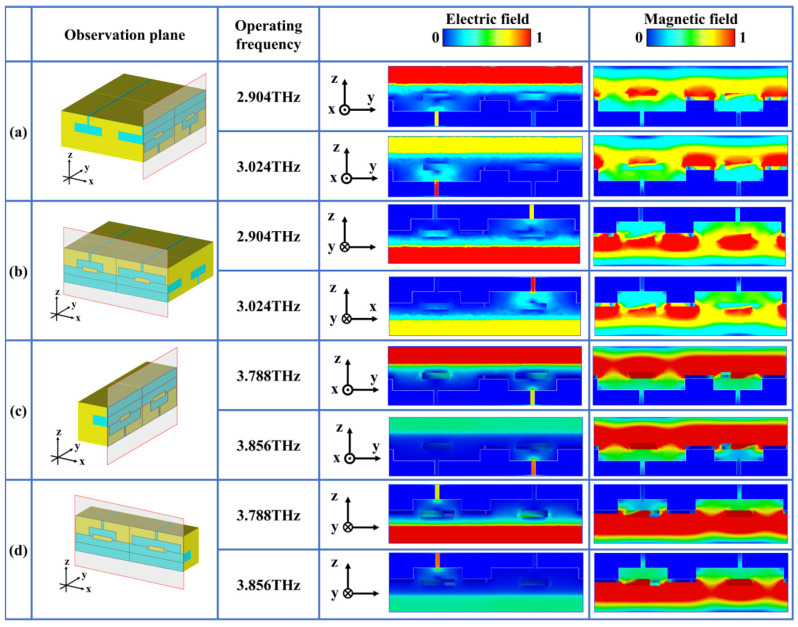
Distributions of electric and magnetic fields within the proposed checkerboard HR metasurface, in view of (**a**,**b**) the *yoz* plane and (**c**,**d**) the *xoz* plane.

Figure 3 shows the normalized electric and magnetic field distributions at four CPA frequency points in the *yoz* and *xoz* observation planes, respectively. From the electric field distribution, it can be observed that the electric field intensity in the slit is much greater than that inside the cavity. This demonstrates that when electromagnetic waves illuminate the surface of the unit, they transmit through the slit into the interior of the cavity, with relatively uniform electric field intensity during propagation. From the magnetic field distribution, we can see that at 2.904 THz, regions with higher magnetic field intensity are primarily located within the cavity, while the magnetic field intensity around the central metal block is relatively weak. At 3.024 THz, a significant difference is observed compared to 2.904 THz, in that the magnetic field intensity around the central metal block substantially increases. Additionally, we can observe that at 3.024 THz and 2.904 THz, the wider cavity primarily operates, while at 3.788 THz and 3.856 THz, the narrower cavity is dominant. The two cavities of different widths exhibit strong independence, which can be leveraged when adjusting the frequencies for dual-band CPA.

**Figure 4:**
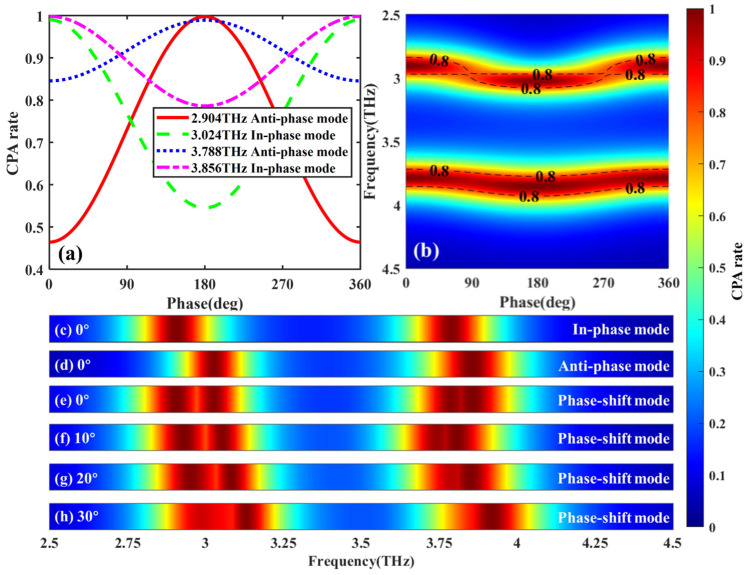
(**a**) Absorption characteristics under in-phase and anti-phase excitation modes. (**b**) Relationship between CPA rate and dual-incident phase difference at the frequency of maximum CPA rate. (**c**–**e**) Bandwidth comparison across different modes. (**f**–**h**) Bandwidth comparison across different incident angles.

As observed in Figure 4a,b, when the phase difference of the incident waves changes, the CPA rates of the two modes vary between their maximum and minimum values. For the in-phase mode at 3.024 THz, the CPA rate changes from 54.52% to 99.00%, while for the anti-phase mode at 2.904 THz, the CPA rate ranges from 46.42% to 99.01%. Direct modulation of the absorption frequency can be achieved via phase difference-assisted control, enabling continuous shifting of the absorption peaks corresponding to both the in-phase and anti-phase CPA modes while maintaining an absorption rate exceeding 80%. This enables continuous shifting of the absorption peaks associated with both the in-phase and anti-phase CPA modes, thereby bridging the originally isolated resonant frequencies into a broadened continuous absorption band. Therefore, unlike conventional HR absorbers, where resonance frequencies are fixed once the geometry is defined, the proposed metasurface enables phase-assisted frequency reconfiguration. This mechanism allows discrete resonant frequencies to be dynamically shifted and interconnected, forming a broadened and reconfigurable absorption band. As shown in Figure 4c–e, under phase difference-assisted control, compared with the sole use of the in-phase and anti-phase modes, the bandwidth corresponding to an absorption rate of 80% in the phase-shifted mode increases from 0.26 THz to 0.448 THz, representing an enhancement of 72.3%. This demonstrates that the proposed structure not only supports multiple resonant frequencies but also efficiently transforms the conventional point-frequency response characteristic of HR absorbers into a dynamically shifted absorption band with enhanced bandwidth without the need for multilayer stacking or increased structural thickness. Furthermore, Figure 4e–h delineate the absorption performance of the phase-shifted mode across incident angles spanning 0° to 30°. Notably, within this angular range, the bandwidth sustaining an absorption efficiency exceeding 80% consistently remains above 0.42 THz. This result substantiates the robust angular stability of the proposed checkerboard HR metasurface. Moreover, within the extended incident angle range of 30° to 60°, the absorption peak associated with CPA progressively diminishes with increasing angle, falling below 80% at 60° incidence. Concurrently, the central frequency of the absorption peak exhibits the identical shift pattern observed in the 0°–30° range—specifically, a consistent low-frequency shift with increasing angle, where the shift magnitude shows an approximately linear relationship with the incident angle. These findings objectively delineate the performance boundary of the metasurface absorber at large angles while underscoring the design’s exceptional stability within conventional operational ranges of less than 30°.

**Figure 5:**
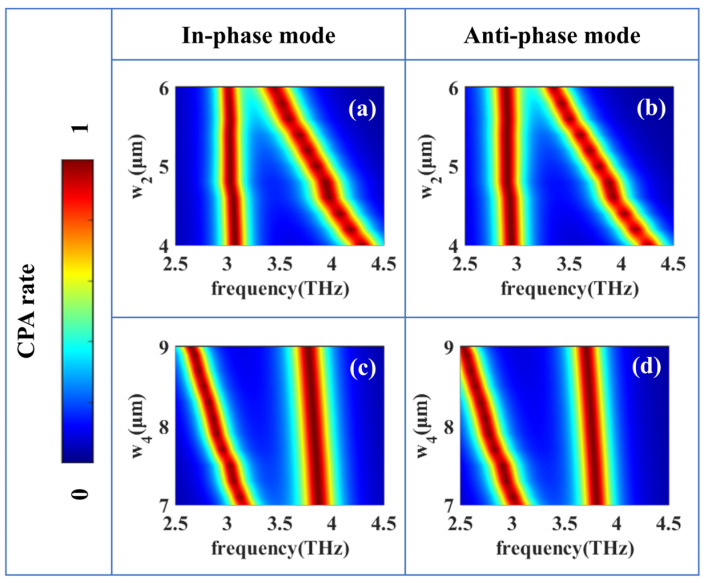
Structural decoupled tunability of the dual-band and dual-mode CPA responses of the checkerboard HR metasurface when adjusting $w_{2}$ while keeping $w_{4}$ constant (**a**,**b**), and adjusting $w_{2}$ while keeping $w_{4}$ constant (**c**,**d**).

Figure 5 continues to investigate the structural decoupled tunability of the dual-band CPA responses in the checkerboard HR metasurface. To further clarify the independence of the two absorption bands, a parametric study was conducted. Specifically, when varying *w*_2_ while keeping *w*_4_ fixed, only the lower-frequency absorption peak shifts significantly, whereas the higher-frequency peak remains nearly unchanged. Similarly, tuning *w*_4_ primarily affects the higher-frequency band with negligible impact on the lower-frequency band. These results indicate that, despite the physical proximity, the residual inter-cavity coupling is relatively weak and does not compromise the independent tunability of the two resonances. By varying the cavity widths $w_{2}$ and $w_{4}$ independently while keeping all other structural parameters fixed, we show that the two absorption frequencies can be controlled in an almost completely isolated manner. As shown in Figure 5a,b, when $w_{2}$ = 5 μm remains constant and only $w_{2}$ is increased from 7 μm to 9 μm, just the higher-frequency absorption peak experiences a pronounced frequency shift, whereas the lower-frequency peak remains essentially unchanged. For the in-phase mode, the low-frequency absorption peak is tuned by 0.060 THz while the high-frequency peak shifts by 0.952 THz. For the anti-phase mode, the low-frequency peak is tuned by 0.041 THz, and the high-frequency peak shifts by 0.916 THz, resulting in a coupling strength of less than 5%. CPA performance is retained throughout the tuning process. Conversely, Figure 5c,d demonstrate that when $w_{4}$ = 7.5 μm is held fixed, and only $w_{4}$ is adjusted from 4 μm to 6 μm, the lower-frequency absorption peak shifts significantly, while the higher-frequency peak stays effectively constant. For the in-phase mode, the low-frequency absorption peak is tuned by 0.584 THz while the high-frequency peak shifts by 0.082 THz. For the anti-phase mode, the low-frequency peak is tuned by 0.524 THz, and the high-frequency peak shifts by 0.077 THz, yielding a coupling strength of less than 15%. These results confirm that the checkerboard HR cavities contribute to independently tunable resonant channels, enabling one absorption frequency to be precisely controlled without perturbing the other.

Further comparison with previous HR architectures, as summarized in Table 1, has been carried out to demonstrate the design flexibility of the proposed checkerboard HR metasurface. Helmholtz resonance is a universal physical mechanism spanning both acoustics and electromagnetics. These two domains share identical resonance principles, coupling behaviors, and multi-mode modulation rules, thereby exhibiting high comparability in structural topology, decoupling characteristics, and multi-band/dual-mode operational mechanisms. Clearly, the asymmetric transmission characteristics induced by the interleaved Helmholtz resonator sublattices with the intrinsic forward–backward transmission imbalance redistributes the scattering phase and amplitude between the counter-propagating channels, enabling the CPA conditions to be independently satisfied under both in-phase and anti-phase modes. Meanwhile, the planar checkerboard configuration spatially separates resonant pathways associated with distinct HR geometries, effectively suppressing mutual coupling and mode hybridization, further fulfilling the dual-mode CPA with highly decoupled operating frequencies at different dual bands. More importantly, by actively tuning the relative phase difference of coherent excitation, the absorption peaks corresponding to each CPA channel can be continuously shifted while maintaining high absorption levels, effectively transforming the intrinsic point-frequency response of conventional HR absorbers into an expanded and tunable absorption band. This phase-assisted spectral modulation provides an additional degree of freedom that is absent in most previously reported HR-based CPA designs.

## 3. Conclusions

In conclusion, we have proposed and demonstrated a planar checkerboard metasurface composed of interleaved HR units with distinct geometrical parameters, enabling decoupled dual-band CPA under both in-phase and anti-phase excitations. Full-wave simulations verify absorption efficiencies exceeding 99% at four target frequencies, corresponding to two pairs of coherent resonant modes. Meanwhile, with the aid of phase modulation, bandwidth extension with absorption efficiency over 80% can be achieved in comparison with the single-mode counterpart. Compared with conventional multilayer or cascaded HR absorbers, the proposed approach offers reduced thickness, enhanced spectral stability, and improved robustness with respect to excitation conditions. This work provides a compact and versatile solution for terahertz coherent absorption and offers a promising platform for developing low-profile, frequency-selective, and integration-ready photonic and electromagnetic devices.

## Figures and Tables

**Table 1:** Comparison between the proposed HR metasurface and related studies.

Refs.	UPA/ CPA	Profile	Freq.	Absorption rate	Structural Decoupled Tunability
[11]	UPA	0.11$\lambda_{h}$	Dual peak	>98%	Yes
[14]	Single-mode CPA	0.23$\lambda_{h}$	Dual peak	>90%	No
[15]	UPA	0.11$\lambda_{h}$	Dual peak	>60%	No
[16]	UPA	0.05$\lambda_{h}$	Multi-peak	>60%	No
[17]	UPA	0.11$\lambda_{h}$	Dual peak	>80%	Yes
This work	Dual-mode CPA	0.07$\lambda_{h}$	Four peaks/ dual band	>99%/ >80%	Yes

## Data Availability

Data will be made available upon request.
